# Phylogeographic patterns of *Merodon* hoverflies in the Eastern Mediterranean region: revealing connections and barriers

**DOI:** 10.1002/ece3.2021

**Published:** 2016-03-04

**Authors:** Gunilla Ståhls, Ante Vujić, Theodora Petanidou, Pedro Cardoso, Snezana Radenković, Jelena Ačanski, Celeste Pérez Bañón, Santos Rojo

**Affiliations:** ^1^ Zoology UnitFinnish Museum of Natural HistoryUniversity of HelsinkiPO Box 1700014HelsinkiFinland; ^2^ Department of Biology and EcologyUniversity of Novi SadTrg Dositeja Obradovića 221000Novi SadSerbia; ^3^ Department of GeographyLaboratory of Biogeography & EcologyUniversity of the Aegean81100MytileneGreece; ^4^ BioSense InstituteUniversity of Novi SadDr Zorana Đinđića 121000Novi SadSerbia; ^5^ Department of Environmental Sciences & Natural Resources/Research Institute CIBIOUniversity of AlicanteApdo 99E‐03080AlicanteSpain

**Keywords:** Aegean archipelago, haplotype diversity, *Merodon*, mtDNA COI, phylogeography

## Abstract

We investigated the phylogeographic patterns of *Merodon* species (Diptera, Syrphidae) in the Eastern Mediterranean. Ten species were sampled on five different islands and mainland sites as a minimum. All samples were screened for their mtDNA COI barcode haplotype diversity, and for some samples, we additionally generated genomic fingerprints. The recently established zoogeographic distribution categories classify these species as having (1) Balkan distribution; (2) Anatolian distribution; (3) continental areas and large islands distribution; and (4) with wide distribution. The ancestral haplotypes and their geographical localities were estimated with statistical parsimony (TCS). TCS networks identified as the ancestral haplotype samples that originated from localities situated within the distributional category of the species in question. Strong geographical haplotype structuring was detected for many *Merodon* species. We were particularly interested to test the relative importance of current (Aegean Sea) and past Mid‐Aegean Trench) barriers to dispersal for *Merodon* flies in the Aegean. We employed phylogenetic *β*‐diversity (P*β*_total_) and its partition in replacement (P*β*_repl_) and richness difference (P*β*_rich_) to test the importance of each explanatory variable (interisland distance, MAT, and island area) in interisland differences using partial Mantel tests and hierarchical partitioning of variation. *β*‐Analyses confirmed the importance of both current and past barriers to dispersal on the evolution of group. Current interisland distance was particularly important to explain the replacement of haplotypes, while the MAT was driving differences in richness of haplotypes, revealing the MAT as a strong past barrier whose effects are still visible today in the phylogenetic history of the clade in the Aegean. These results support the hypothesis of a highly restricted dispersal and gene flow among *Merodon* populations between islands since late Pleistocene. Additionally, patterns of phylogeographic structure deduced from haplotype connections and ISSR genome fingerprinting data revealed a few putative cases of human‐mediated transfers of *Merodon* spp.

## Introduction

Hoverflies (Diptera, Syrphidae) constitute an important pollinator group standing in importance next only to bees (Hymenoptera, Apoidea). The group comprises some 6000 classified species worldwide (Brown 2009; Thompson 2013). They are vigorous flyers with an exceptional ability to hover motionless in the air. The majority of the adults visit flowers to ingest pollen and nectar as source of protein and energy. Many species are excellent mimics of stinging hymenopterans, and some groups even exhibit same behavior patterns as their hymenopteran models (Penney et al. 2014). In contrast, larvae can be saprophagous, phytophagous, or predators. Saprophagous larvae can be found on a diverse array of organic substrates such as decaying wood, dung, and fungi. Predators often rely on aphids and other soft‐bodied Hemiptera (see Rojo et al. 2003). Phytophagous species mine in plant leaves, stems, and bulbs (e.g., Reemer and Rotheray 2009; Van Zuijen and Nishida 2011; Reemer 2013). The last functional group of species is found only on a few genera of the subfamily Eristalinae, viz. *Cheilosia* (tribe Rhingiini)*, Eumerus,* and *Merodon* (tribe Merodontini) (e.g., Hurkmans 1993; Stuke 2000 and references therein).

Hoverflies of the genus *Merodon* are distributed in the Palearctic and Afrotropical biogeographic regions, and occur mainly in areas with a Mediterranean climate regime (Hurkmans 1993). In Europe, the genus has a predominantly Mediterranean distribution and is the largest genus of European hoverflies with 120 species (Speight 2014). Only in Greece, 68 species have been recorded (Vujić et al. 2016), and the estimate of the species number for Turkey is expected to exceed 60 (Vujić et al. 2011, unpubl. data). All species of the genus are expected to share the same larval feeding habit as miners of bulbs and underground storage organs of geophyte plants mainly of the families Liliaceae, Amaryllidaceae, and Hyacinthaceae. The relationship between the immature stages of *Merodon* species and their specific host plants, however, remains unknown for more than 90% of the taxa (Hurkmans 1993; Ricarte et al. 2008; Andrić et al. 2014).

Recent taxonomic studies on *Merodon* in the Eastern Mediterranean and Anatolia have resulted in descriptions of multiple new *Merodon* taxa (Radenković et al. 2011; Popov 2009; Vujić et al. 2007; 2011, 2013, in preparation). During the course of these studies and more recently as part of the POL‐AEGIS project (Petanidou et al. 2013), we have obtained extensive and detailed information on the occurrence and distributions on probably all *Merodon* spp. in Greece, and specifically on species occurring in the Aegean region. We have also gathered occurrence data for the species from chosen localities situated on coastal areas adjacent to seaboard islands of Asia Minor and the Greek mainland (see Vujić et al. 2016 for details).

**Figure 1:**
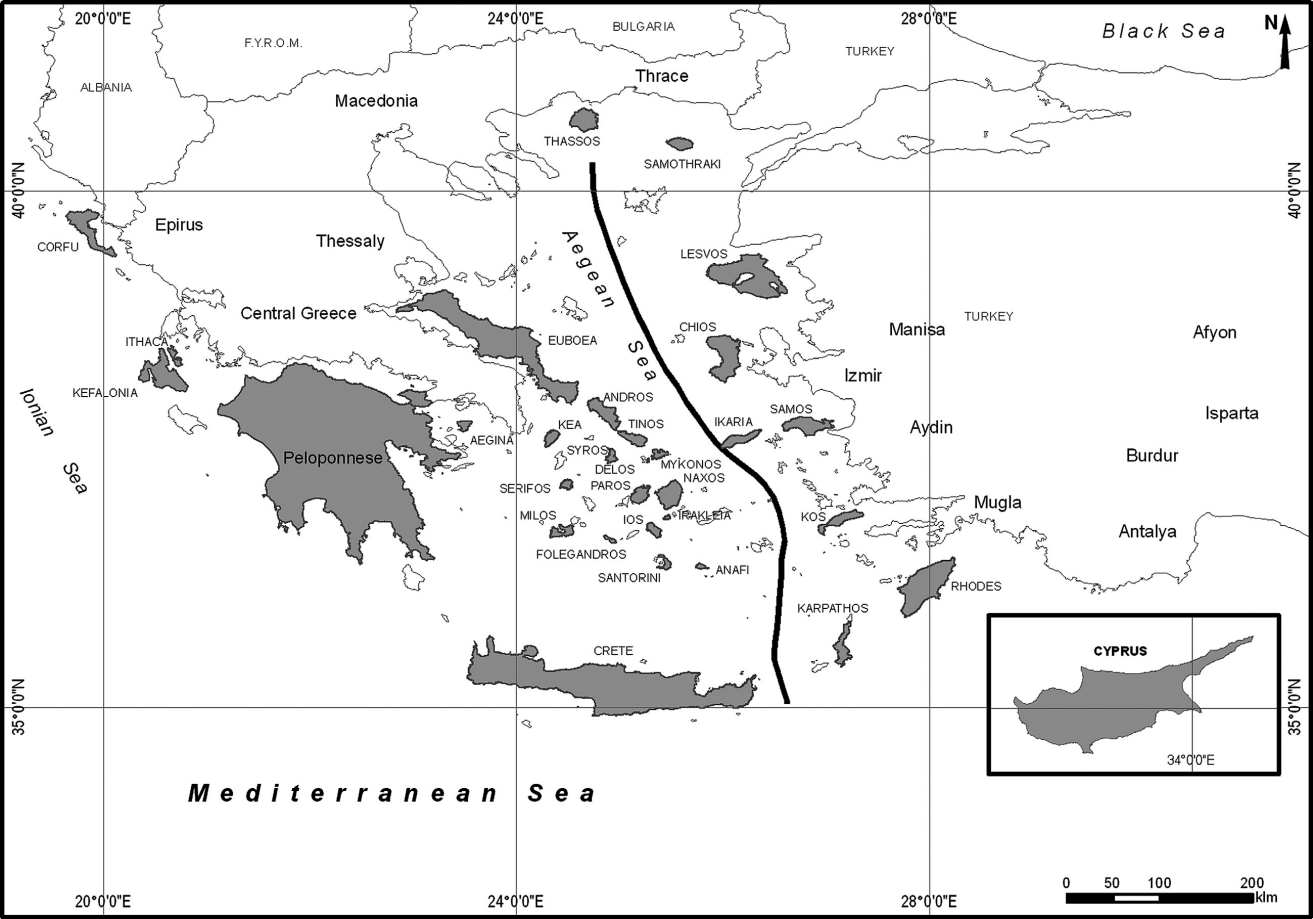
Study area with islands sampled for *Merodon* spp. for molecular work shaded in gray, sampled mainland sites indicated with filled circles, Mid‐Aegean Trench (dividing line) indicated.

The Paleogeographic history of the Aegean region is long, multifaceted, and complex. In short, one of the main geological events was the formation of the Mid‐Aegean Trench (MAT) or dividing line formed between ca 12 and 9 Mya, which initiated the contemporary configuration of the Aegean region (Fig. 1). Among all, MAT is considered as a geographical (deep‐sea) barrier between the Anatolian and Balkan Peninsulas (Dermitzakis and Papanikolaou 1981; Lymberakis and Poulakakis 2010). After the Aegaeis land collapse followed by the Messinian Salinity crisis (5.96–5.33 Mya), it was the refilling of the Mediterranean Basin from the Atlantic, which separated for the first time the Aegean Islands E and W of the MAT (Krijgsman et al. 1999; Duggen et al. 2003). Cycles of connection and fragmentation of the Aegean Islands from one another and their adjacent mainland resulted from changes in sea levels during Pliocene and Pleistocene (e.g., Dermitzakis and Papanikolaou 1981). All these events have greatly influenced the historical biogeography of plants and animals in the area as shown in multiple studies (e.g., Sfenthourakis and Legakis 2001; Bittkau and Comes 2005, 2009; Allegrucci et al. 2009; Georghiou and Delipetrou 2010; Lymberakis and Poulakakis 2010; Simaiakis et al. 2012). The Aegean archipelago is thus an interesting area for phylogeographic studies for tracing both the ancient and more recent biogeographic events.

Phylogeography is the phylogenetic analysis of geographically contextualized genetic data and emphasizes the historical aspects of the contemporary spatial distributions of gene lineages (Avise et al. 1987; Avise 2000, 2007). MtDNA COI barcode sequences (Hebert et al. 2003) were extensively used in phylogeographic studies of invertebrates, especially for insects. A recent review by Poulakakis et al. (2015) evaluated more than 100 phylogeographic articles on 76 animal genera (30 invertebrates and 46 vertebrates), many applying the COI barcode sequences, that have been published so far for the Aegean region, but none of the studies concerned flies (Diptera). The use of flies as model organisms in phylogeographic studies has been very limited. Many flying insects are relatively mobile, and the generally high dispersal abilities of flies predict minimal or nonexistent phylogeographic structuring (Avise 2000). Indeed, dispersal ability has been found to be the decisive factor that was predictive of the degree of genetic diversity (and the speciation patterns) in various lineages of tenebrionid beetles distributed in the Aegean archipelago (Papadopoulou et al. 2009). Tenebrionidae species with flight ability showed a low degree of genetic geographical structuring for the tested mtDNA genes, while the converse situation was found for the flightless species.

The Aegean area constitutes an interesting melting pot of mainly Balkan and Anatolian faunal elements, together with elements from the Ethiopian region; among all, it harbors a high diversity of *Merodon* species including species endemic to the region (Petanidou et al. 2011; Radenković et al. 2011; Vujić et al. 2016). The region also shows an exceptional richness of geophyte host plants (Bazos 2005; Dimopoulos et al. 2013).

Despite the adult flies being medium‐ to large‐sized (1–2.5 cm) insects with good flight ability, our observations show that *Merodon* species are tightly connected to the habitats of their larval geophyte host plants, and thus, they are more residential in their habitats than many other hoverfly species (Vujić et al. 2012, 2016; Andrić et al. 2014). Therefore, we predict that the *Merodon* taxa have potential as objects for phylogeographic studies and have identified the Eastern Mediterranean region as an optimal study region.

The aim of this study was to describe the haplotype distribution patterns using mtDNA COI barcode sequences for a chosen set of *Merodon* species in the wider Aegean area. We were particularly interested in testing the relative importance of the current (interisland distance) and past (Mid‐Aegean Trench) as a dispersal barrier to these taxa. Our second aim was to explore the congruence of the phylogeographic estimates of locations of ancestry established in this study based on the mtDNA COI haplotypes and statistical parsimony analyses using TCS with the zoogeographic distributional categories for *Merodon* species established by Vujić et al. (2016). For this aim, we have also tested Inter‐Simple Sequence Repeats data (genomic fingerprints) as a potential source of intraspecific variation for some of the study species.

## Materials and Methods

### *Merodon* distributions, taxon choice, and specimen sampling

Vujić et al. (2016) compiled species distribution data from southeastern Europe and western Turkey for 78 species of *Merodon*. The data collection was based on recent collections of *Merodon* from the study islands and the surrounding mainland areas (Greek and Anatolian Peninsulas) during the period 2003–2014, as well as multiple visits to insect collections of main European Natural History Museums to re‐identify and record existing (mainly old) *Merodon* specimens. The resulting dataset consisted of a total of 12,108 obtained *Merodon* specimens or specimen records with locality and other data. Of these data, 53 *Merodon* species were registered for the wider Aegean area, and of these, we chose ten study species. The choice was made on the basis of the main characteristics of their distributional patterns also considering their abundance and occurrence of each species in at least five study islands/mainland sites (Table 1).

**Table 1:**
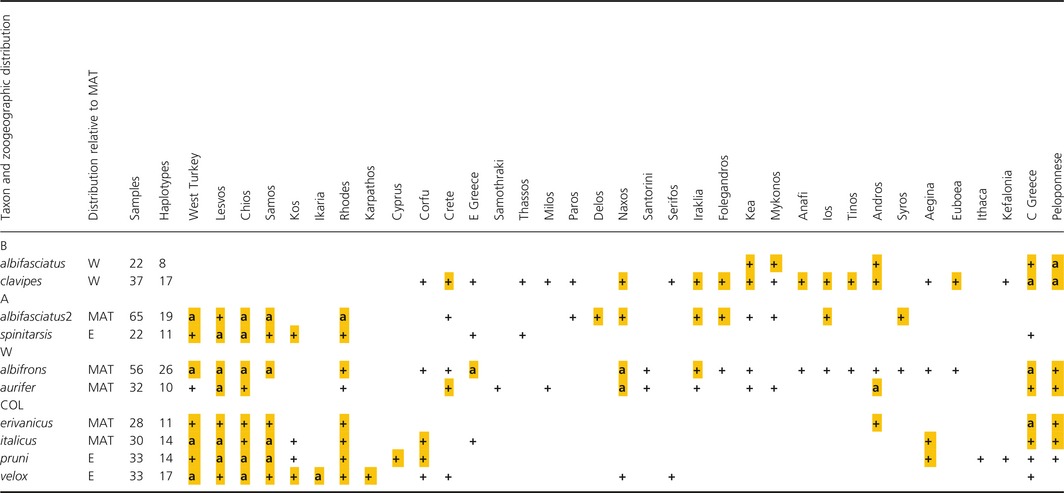
List of included species with zoogeographic classification of *Merodon* species in Eastern Mediterranean Islands and mainland regions sensu Vujić et al. (2016). Presence of species on respective island or mainland is indicated with a “+,” the islands or mainland regions from which specimens were obtained for molecular study are indicated in color and the ancestral haplotype localities are indicated with an “**a**.” The first column gives the total number of samples obtained for mtDNA COI sequencing for each species. A, Anatolia; B, Balkan; W, Wide; COL, Continental and Large Island distributions. The second column gives the distribution of the species relative to the Mid‐Aegean Trench (MAT). W, distributed only West of MAT; E, distributed only East of MAT; MAT, distributed across the MAT. The third column lists the total number of haplotypes registered for each study taxon.

The selected ten study species belong to four different distributional categories as outlined by Vujić et al. (2016), two species have a Balkan distribution (distributed over the Balkans and Eastern Mediterranean Islands, but not in the Anatolian Peninsula), two Anatolian (present in the Anatolian Peninsula and on the Eastern Mediterranean Islands, but absent from the Balkan Peninsula), four are distributed in continental areas and large islands in the Aegean region (present on both peninsulas and some of the islands larger than 420 km^2^, e.g., Corfu, Naxos, Lesvos, Chios, Samos, Rhodes, Cyprus, Crete, and Peloponnese), and two are widespread in the Aegean and beyond (distributed over both peninsulas, on Eastern Mediterranean Islands of all sizes, and elsewhere in the Mediterranean area). Five of the included taxa are distributed across the MAT in the Aegean Sea, two occur only west of MAT, and three species only East of MAT (Table 1).

**Figure 2:**
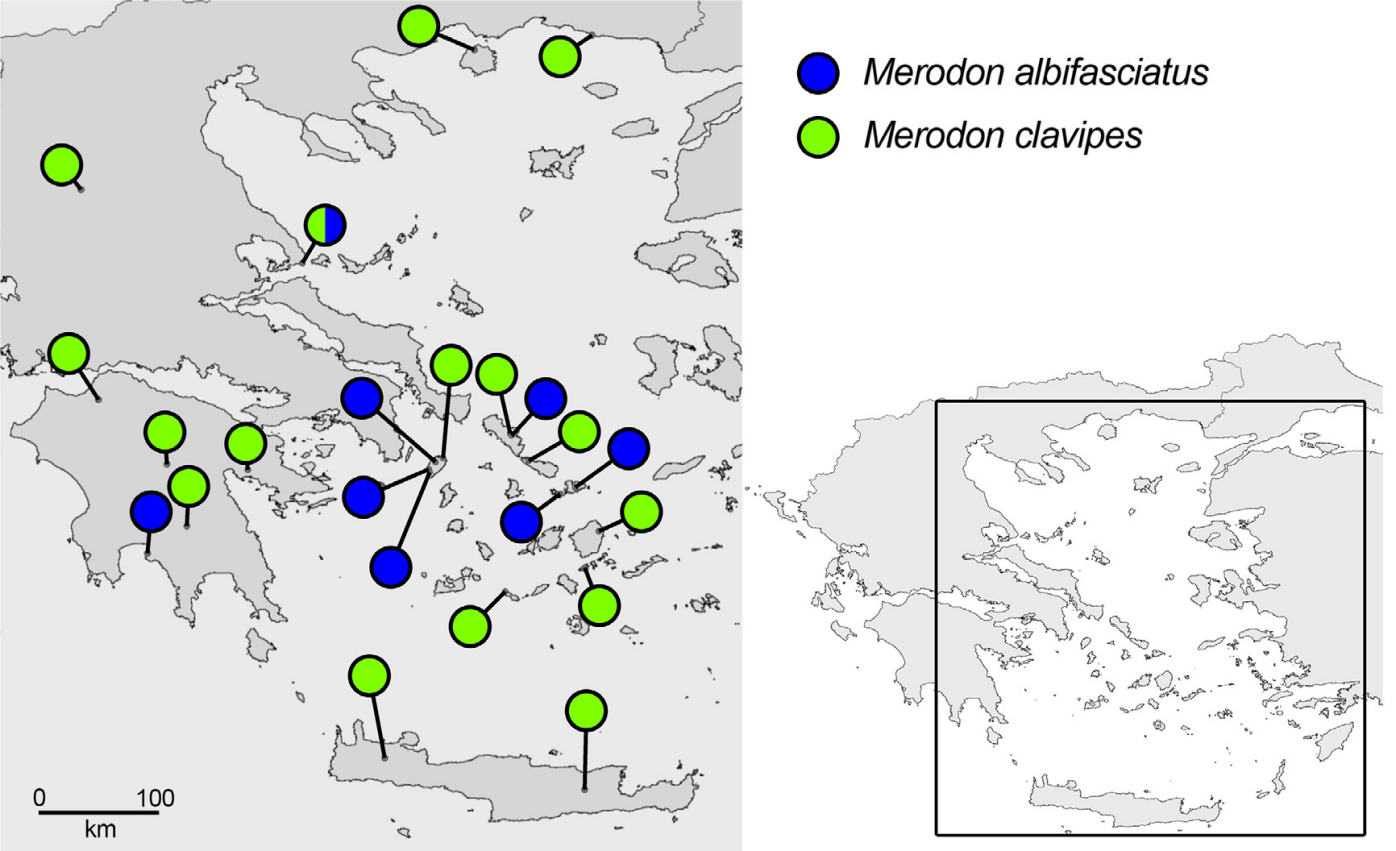
Sampled localities in the Aegean Islands and continental areas. *Merodon albifasciatus* and *M. clavipes*.

**Figure 3:**
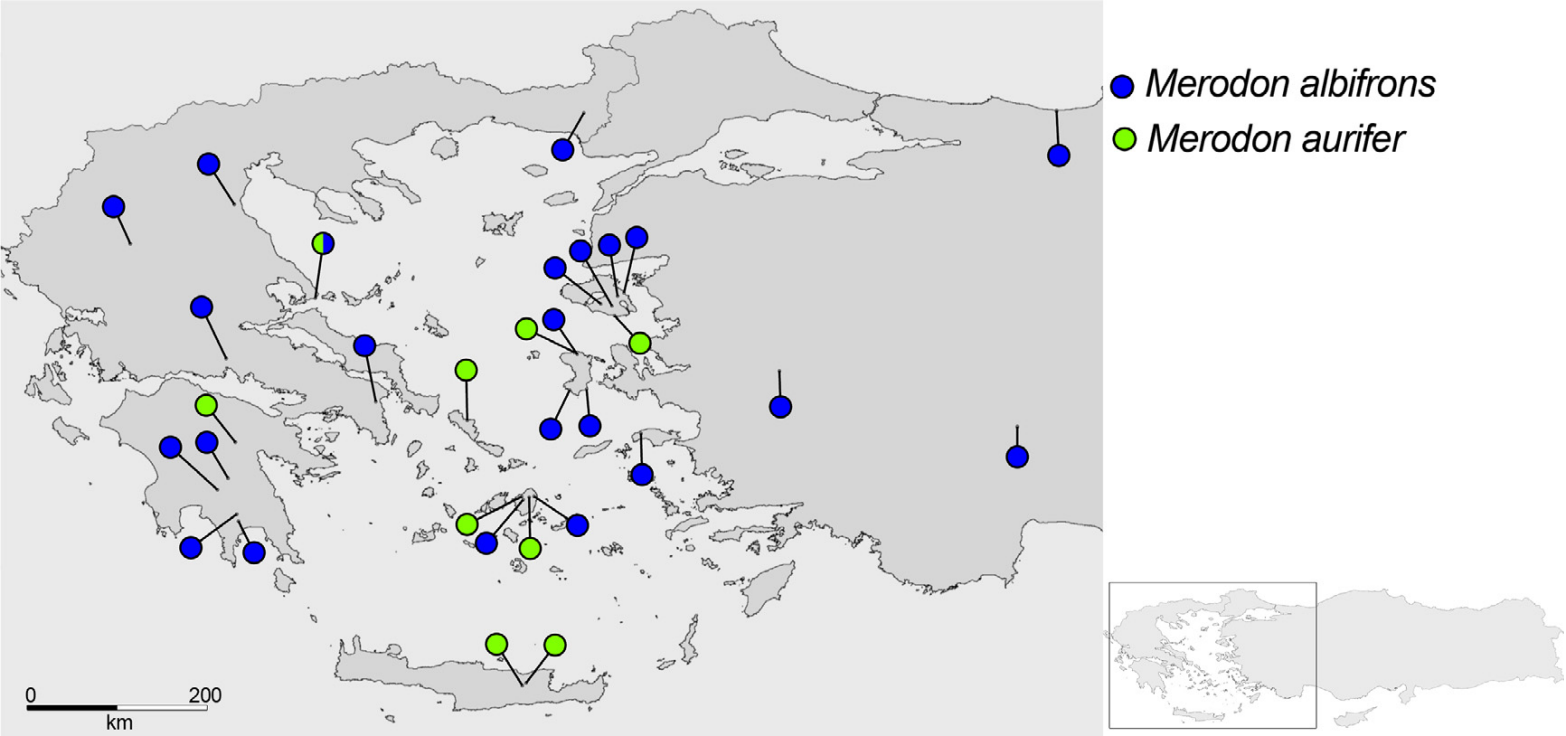
Sampled localities in the Aegean Islands and continental areas. *Merodon albifrons* and *M. aurifer*.

**Figure 4:**
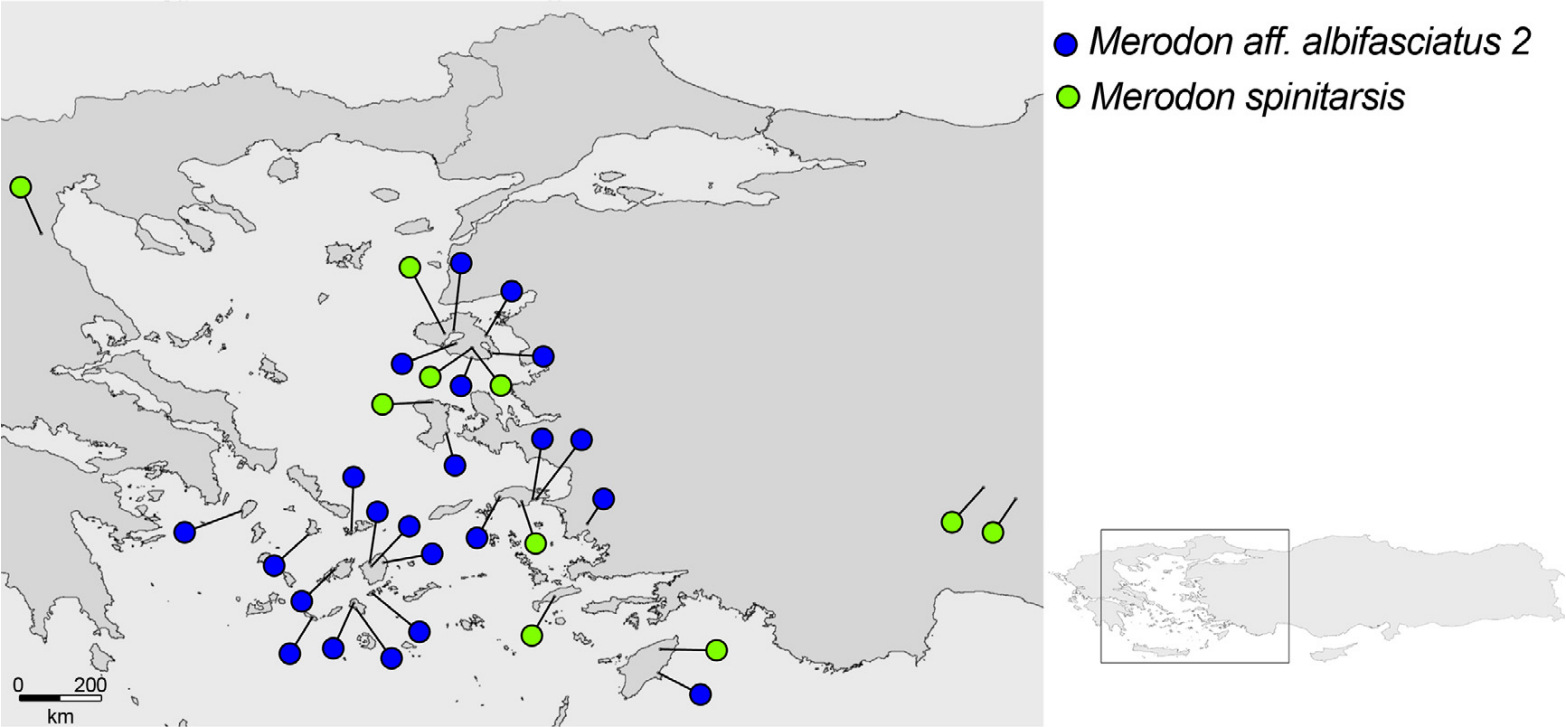
Sampled localities in the Aegean Islands and continental areas. *Merodon* aff*. albifasciatus*2 and *M. spinitarsis*.

**Figure 5:**
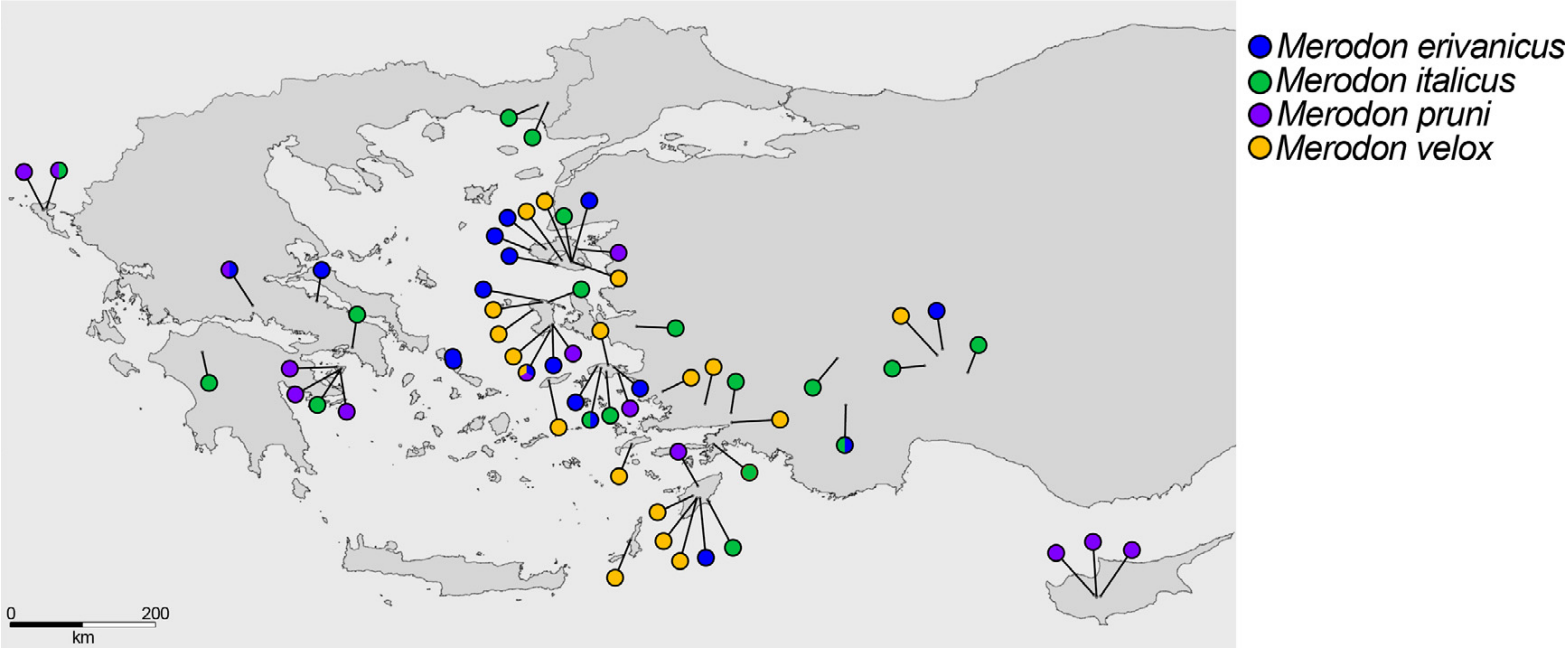
Sampled localities in the Aegean Islands and continental areas. *Merodon erivanicus*,*M. italicus*,*M. pruni*, and *M. velox*.

When sampling specimens for molecular analysis, we employed hand netting as this sampling method is the most efficient for this group of insects. We aimed at including about five samples of each species from each island, but we could not obtain this sample size for all localities. Sampling was carried out from spring 2010 to autumn 2014 on 32 islands in the Aegean and on several sites on the Greek and Anatolian mainland areas adjacent to the surveyed islands (Figs. 2, 3, 4, 5). Sampling was repeated either systematically or randomly on different islands and areas as described in detail by Vujić et al. (2016).

Each specimen subjected to molecular analysis was labeled as a DNA voucher specimen and is deposited in FSUNS (Insect collection of the Faculty of Science, University of Novi Sad, Serbia), MZH (Insect collection of the Zoology unit, Finnish Museum of Natural History, Helsinki, Finland), or the Melissotheque of the Aegean (insect collection of the University of the Aegean). All *Merodon* specimens were registered in the database of the Melissotheque of the Aegean. The specimen and locality data of newly sampled material and of previously obtained specimens including the GenBank accession numbers for the COI barcode are listed in online Supplementary information Table S1.

### Sampling localities of study species

Sampled localities of the Balkan species *Merodon albifasciatus* (Macquart) and *M. clavipes* (Fabricius) are shown in Figure 2, species with Wide distribution *Merodon aurifer* Loew and *M. albifrons* Meigen are shown in Figure 3, the sampled localities of Anatolian species *Merodon* aff. *albifasciatus*2 sensu Vujić et al. (2016) (hereafter *Merodon albifasciatus*2) and *Merodon spinitarsis* Paramonov in Figure 4 *,* and species distributed on Continental areas and large islands being *Merodon erivanicus* Paramonov, *M. italicus* Rondani, *M. pruni* (Rossi) and *M. velox* Loew in Figure 5.

### Choice of genetic markers

MtDNA COI that was the marker of choice in this study was used in all hitherto published studies on molecular systematics or evolution on Syrphidae (see references concerning Syrphidae). These studies have shown that the level of nucleotide variability of COI spans both the intraspecific (mainly 3rd codon position mutations) and interspecific levels (also 1st codon position mutations) and higher taxonomic levels, and the marker has been highly informative and congruent with hypotheses based on morphological data (e.g., Vujić et al. 2011, 2012, 2013; Mengual et al. 2015). Altogether, a few cases of inconsistency between morphological and molecular hypothesis have been observed among Syrphidae taxa in studies concerning very closely related taxa. Introgression among closely related taxa or incomplete lineage sorting resulting in haplotype sharing being hypothesized as likely causes in these studies (e.g., Milankov et al. 2009, 2013; Ståhls et al. 2009; Francuski et al. 2014; Haarto and Ståhls 2014). To further assess the presence of genetic polymorphism within the study taxa, the entire nuclear genome was screened using Inter‐Simple Sequence Repeats (ISSR) with a tetra‐nucleotide primer, (GACA)_4,_ and converted into a genomic fingerprint. Such DNA profiles have been used for population genetics or discrimination of sibling taxa (Kehlmaier and Assmann 2009 and references therein).

### Laboratory procedures

DNA was extracted from two legs of the dry, pinned adult fly (using the Nucleospin Tissue DNA extraction kit (Machery‐Nagel, Düren, Germany) following manufacturer's protocols and re‐suspended in 50 *μ*L of ultra‐pure water. The PCR and sequencing protocols follow those described in Ståhls et al. (2009). The Folmer fragment or “barcode fragment” of the 5′ region of COI was amplified using PCR protocols with forward primer LCO (5′‐GCTCAACAAATCATAAAGATATTGG‐3′) and reverse primer HCO (5′‐TAAACTTCAGGGTGACCAAAAAATCA‐3′) (Folmer et al. 1994). Amplified PCR products were electrophoresed on 1.5% agarose gels and treated with Exo‐SapIT (USB Affymetrix, Cleveland, OH) prior to sequencing. Both PCR primers were used for sequencing. The Big Dye Terminator Cycle Sequencing Kit (version 3.1) (Applied Biosystems, Foster City, CA) was used on an ABI 3730 (Applied Biosystems) genetic analyzer at the Sequencing Service Laboratory of the Finnish Institute for Molecular Medicine (www.fimm.fi). The sequences were edited for base‐calling errors and assembled using Sequencher^™^ (version 5.0) (Gene Codes Corporation, Ann Arbor, MI). The ISSR PCRs used a nonanchored tetra‐nucleotide primer (GACA)4. PCR conditions were an initial denaturation of 5 min at 94°C, followed by 35 cycles of 60 sec at 94°C, 60 sec at 52°C, 3 min at 72°C, and a final elongation of 15 min at 72°C as described in Kehlmaier and Assmann (2009). Genomic fingerprints were obtained by running 10 *μ*L of PCR products were run on a 2% agarose gel at 50 Volts for 2.5 h. Five *μ*L of a low range DNA ladder MBI Fermantas (Amherst NY, USA) was simultaneously run in lateral and mid lanes of each gel.

### Phylogenetic trees

The monophyly of the included taxa was tested using maximum‐likelihood (ML) and Neighbor‐Joining (NJ) Saitou & Nei (1987) analyses using software package MEGA vs6 (Tamura et al. 2013). The analyses included in total 361 ingroup sequences of the ten study taxa, and used a species of *Eumerus* (Syrphidae; Merodontini) as outgroup. MEGA vs6 was also used to choose the best‐fit model under the corrected Akaike information criterion (AICc) for ML analysis. Nonparametric bootstrap support values for NJ and ML analyses were also calculated using MEGA with 1000 replicates. NJ analyses were also made separately for the sequence data of taxa *Merodon aurifer*,* M*. *albifasciatus*2, *M. albifrons*,* M. erivanicus*,* M. pruni,* and *M. spinitarsis*, and obtained NJ trees were spatially plotted on a map using the software GenGIS v.2.4.1 (Parks et al. 2013). Country boundaries of Cyprus, Greece, and Turkey were downloaded from Diva‐Gis website (www.diva-gis.org/gdata).

### The relative importance of current and past barriers to dispersal of *Merodon* species

The correlation between interisland distance and haplotype dissimilarity patterns would allow us to infer on the importance of current dispersal barriers posed by the sea to evolutionary processes. Yet, it would not allow perceiving the importance of the MAT as a barrier reflected on the past evolution of species and populations. Furthermore, it is important to distinguish between species or haplotypes that were unable to cross such barriers (either past MAT or current sea) and those that seem to exhibit replacement patterns related with those same barriers (e.g., haplotypes that are replaced by others on different sides of the MAT). Using the Neighbor‐Joining phylogenetic tree built for all 10 species and 147 haplotypes we partitioned total phylogenetic *β*‐diversity (P*β*_total_) into its replacement (P*β*_repl_) and richness difference (P*β*_rich_) components following Cardoso et al. (2014). For a given pairwise comparison between islands, the phylogenetic diversity (PD) as measured by total branch length connecting all haplotypes present among both islands can be divided into three components: (1) the sum of the length of branches common to both islands (their common evolutionary history); (2) the sum of the branch lengths exclusive to the first island; and (3) the sum of the branch lengths exclusive to the second island (2 and 3 reflecting unique evolutionary history). Using these three components, it is possible to partition total difference in (1) replacement of branches and (2) loss or gain of branches of the phylogenetic tree among islands (Cardoso et al. 2014). Phylogenetic *β*‐diversity analyses were made using the R package BAT (Cardoso et al. 2015). To model the importance of current and past barriers on the evolution of the genus *Merodon* in the Aegean, we used Mantel tests to correlate the three beta‐diversity measures with interisland distance (reflecting current barriers) and a variable we called MAT that was coded as 1 if islands were separated by the Mid‐Aegean Trench or 0 if they were on the same side of it. This allowed perceiving if the MAT could explain current patterns beyond what could be inferred from interisland distance alone. In addition, and given that area could by itself cause differences in phylogenetic diversity between islands, this variable was included as an additional factor in the analyses (after log transformation). Partial Mantel tests were also made so that the importance of each variable after accounting for the other two was quantified. Significance of the Mantel statistic *r*_M_ was assessed with 10,000 permutations using the R package ecodist (Goslee and Urban 2007). Finally, to estimate the proportion of total variation in the data that could be explained by each factor independently we used hierarchical partitioning of variation (Chevan and Sutherland 1991). This method measures the improvement in the *R* ^2^ of all models with a given variable compared with the corresponding models without such variable and averages this improvement as the relative, independent, importance of the variable of interest. We used the R package relaimpo (Groemping 2006) for hierarchical partitioning.

### Haplotype network analyses

To compare genetic connections of geographical areas, we constructed haplotype minimum spanning networks of obtained DNA sequences using statistical parsimony (Templeton et al. 1992), as implemented in the software TCS1.21 (Clement et al. 2000) using a 95% connection limit. The method of Templeton et al. (1992) (TCS) has been frequently used for nucleotide sequence data to infer population level genealogies and is the most informative method for exploring the phylogeographic history of a set of organisms when divergences are low (e.g., Schaal et al. 1998; Vilá et al. 1999; Gómez‐Zurita et al. 2000). Haplotype networks use information on inferred mutational steps between the haplotypes and group closely related haplotypes together. The program collapses sequences into haplotypes and calculates the frequencies of the haplotypes in the sample, in addition to calculating haplotype connections. The highest numbers of frequencies and connections are used to estimate the haplotype outgroup probabilities (Posada and Crandall 2001), which correlate with haplotype age (Donnelly and Tavaré 1986; Castelloe and Templeton 1994). We generated separate haplotype networks for ten *Merodon* species of the different distribution patterns.

## Results

### MtDNA COI barcode sequences

The number of analyzed specimens *per* species varied between 22 and 68 (Table 1). The specimen sampling effort resulted in a total of 361 COI barcode sequences (147 haplotypes) obtained for the ten focal species. The barcodes were pruned to a length of 644 bp for all specimens, and no alignment gaps were observed.

### Monophyly of the study taxa

**Figure 6:**
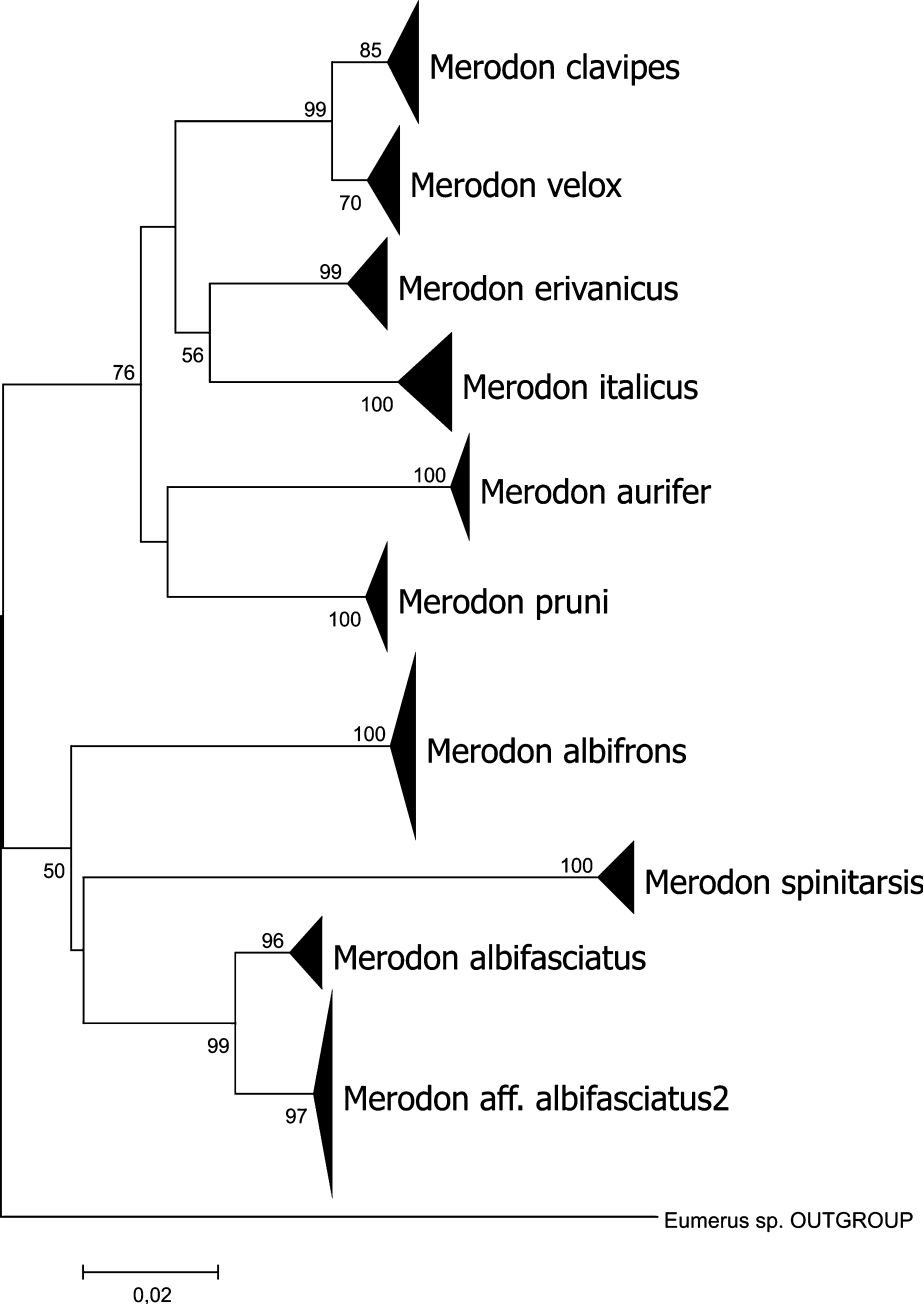
Neighbor‐Joining tree based on the K2P nucleotide substitution model for all COI sequences. Sequence collapsed into triangles for each species (size proportional to the number of sequences), and bootstrap support values ≥50% indicated above branches.

The best‐fit model for the ML analysis was the General Time Reversible (GTR) model + gamma‐distributed rates, while the Kimura 2‐parameter (K2P; Kimura 1980) nucleotide substitution model was used for the NJ analyses. Both NJ and ML analyses recovered all study taxa as monophyletic lineages, the NJ tree is shown in Figure 6, with terminals (sequences) of each taxon collapsed into a triangle for easier visualization.

### The relative importance of current and past barriers to dispersal of *Merodon* species

Overall beta‐diversity (P*β*_total_) could be related with both past and current barriers to dispersal even after accounting for other variables, confirming the importance of the MAT in the current phylogenetic patterns of *Merodon* species (Table 2). Current interisland distance seems, however, to be mostly determinant to the replacement of haplotypes among islands, as indicated by both partial Mantel tests and the larger variation explained by this factor to *β*_repl_. In contrast, *β*_rich_ is mostly determined by the existence of the MAT, probably reflecting the inability of many intraspecific lineages to cross this barrier in the past, which caused a loss of PD (and haplotypes) from one side to the other of the Trench.

**Table 2:** Results of Mantel tests between phylogenetic beta‐diversity measures (total, partition due to replacement of haplotypes and partition due to gain or loss of haplotypes) and distance between islands, MAT region (East or West of the MAT), and area of islands. The results for hierarchical partitioning of variation between variables are also presented as a proportion of explained variation

	Distance	MAT	Area
*β*_total_			
*r*_M_	0.352***	0.340***	0.220*
Partial *r*_M_	0.246*	0.277**	0.181*
Variation	0.086	0.090	0.037
*β*_repl_			
*r*_M_	0.320**	0.121*	0.124
Partial *r*_M_	0.283*	0.034	0.065
Variation	0.090	0.008	0.009
*β*_rich_			
*r*_M_	−0.063	0.148*	0.048
Partial *r*_M_	−0.124	0.177*	0.068
Variation	0.009	0.026	0.003

*r*_M_, Mantel correlation statistic; partial *r*_M_, partial Mantel statistic after accounting for the other variables.

Significance: *P < 0.05; **P < 0.01; ***P < 0.001.

### Congruence of zoogeographic distributional categories with statistical parsimony estimates of ancestral haplotype localities

The COI haplotype distributions of the study species in the wider Aegean area are shown in respective networks (Figs. 7, 8, 9, 10, 11, 12, 13, 14, 15, 16) with ancestral haplotypes indicated as squares, and each haplotype connection line representing one mutational step. The separate NJ analyses for taxa *Merodon albifasciatus*2, *M. aurifer*,* M. pruni,* and *M. spinitarsis* are shown in Figures 17, 18, 19, 20, with NJ trees placed on maps and branching haplotypes (terminal branches) pointing to sampling localities.

**Figure 7:**
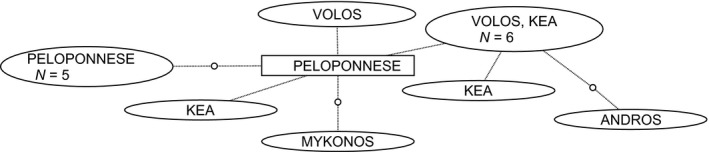
Haplotype network of the Balkan species *Merodon albifasciatus*. In the haplotype networks, the rectangle represents the TCS determination of the haplotype most likely to be ancestral (highest outgroup probability) for the actual set of sequences. Ovals represent different haplotypes, and the size of the rectangles and ovals is proportional to the number of samples sharing the identical haplotype (specimen numbers ≥3 reported in figures). Each connecting line represents one nucleotide change. Intermediate or unsampled haplotypes are visualized with the small open circles on the connecting branches.

**Figure 8:**
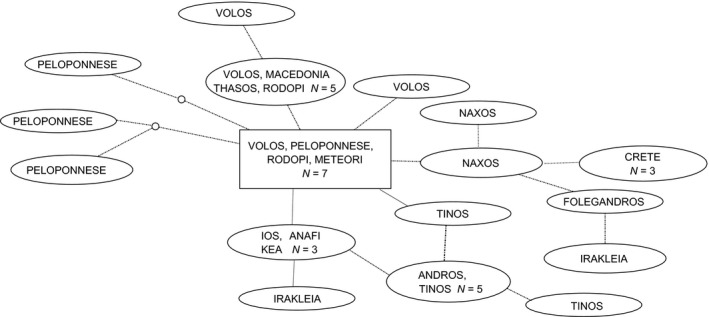
Haplotype network of the Balkan species *Merodon clavipes*.

**Figure 9:**
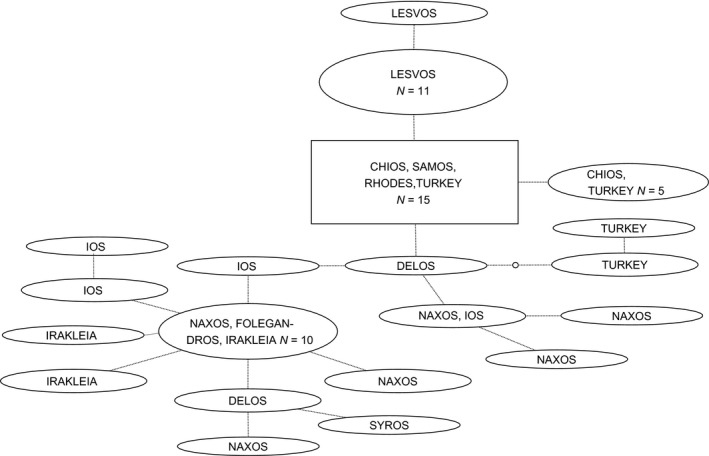
Haplotype network of the Anatolian species *Merodon* aff. *albifasciatus*2.

**Figure 10:**
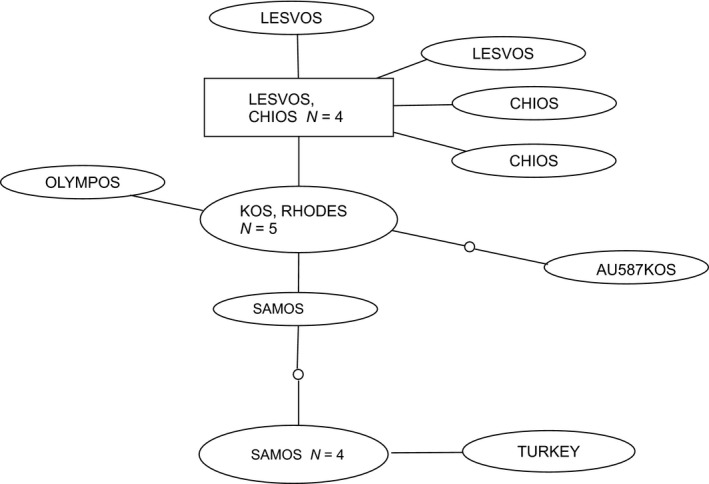
Haplotype network of the Anatolian species *Merodon spinitarsis*.

**Figure 11:**
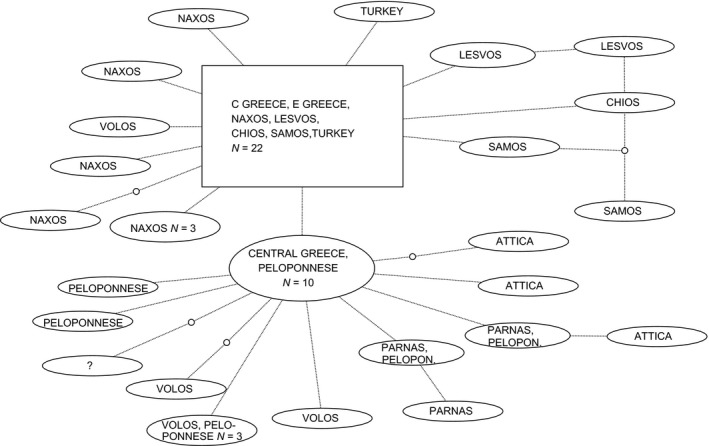
Haplotype network of the Wide distribution species *Merodon albifrons*.

**Figure 12:**
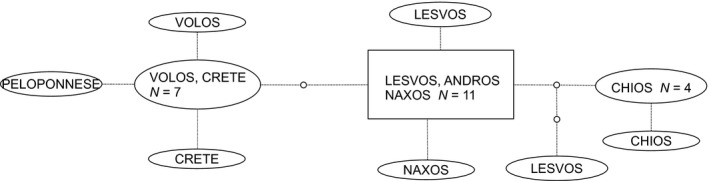
Haplotype network of the Wide distribution species *Merodon aurifer*.

**Figure 13:**
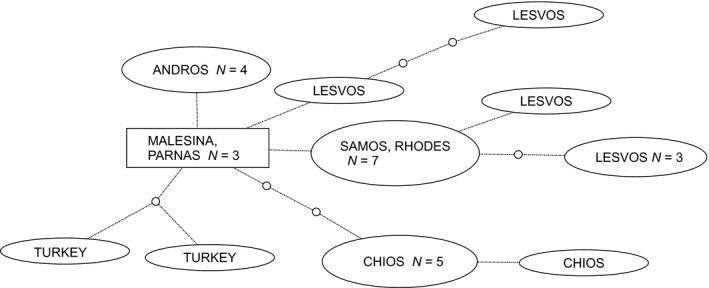
Haplotype network of the Continental and large island distributed species *Merodon erivanicus*.

**Figure 14:**
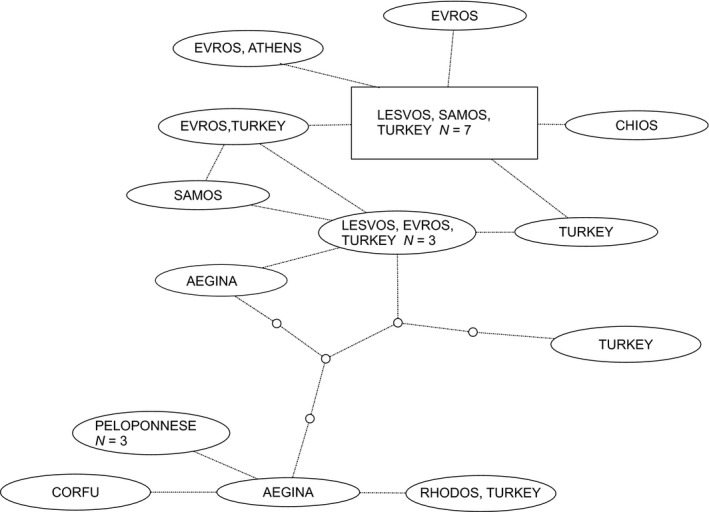
Haplotype network of the Continental and large island distributed species *Merodon italicus*.

**Figure 15:**
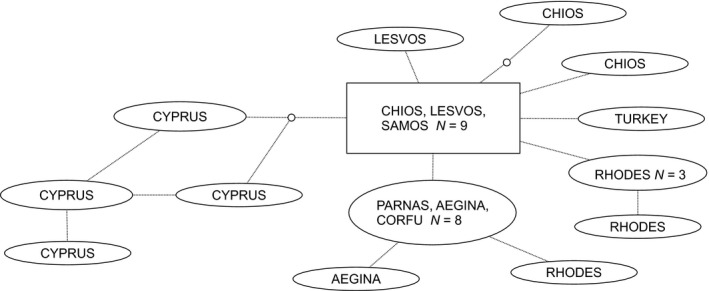
Haplotype network of the Continental and large island distributed species *Merodon pruni*.

**Figure 16:**
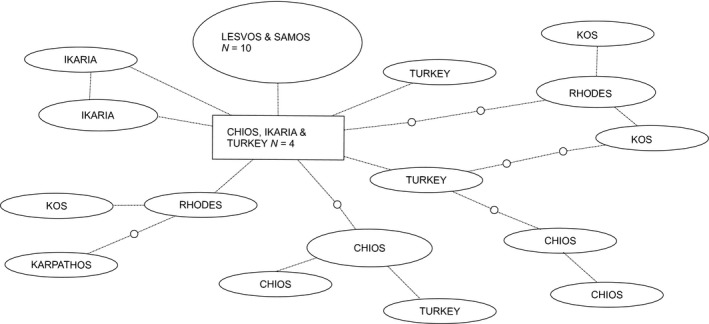
Haplotype network of the Continental and large island distributed species *Merodon velox*.

**Figure 17:**
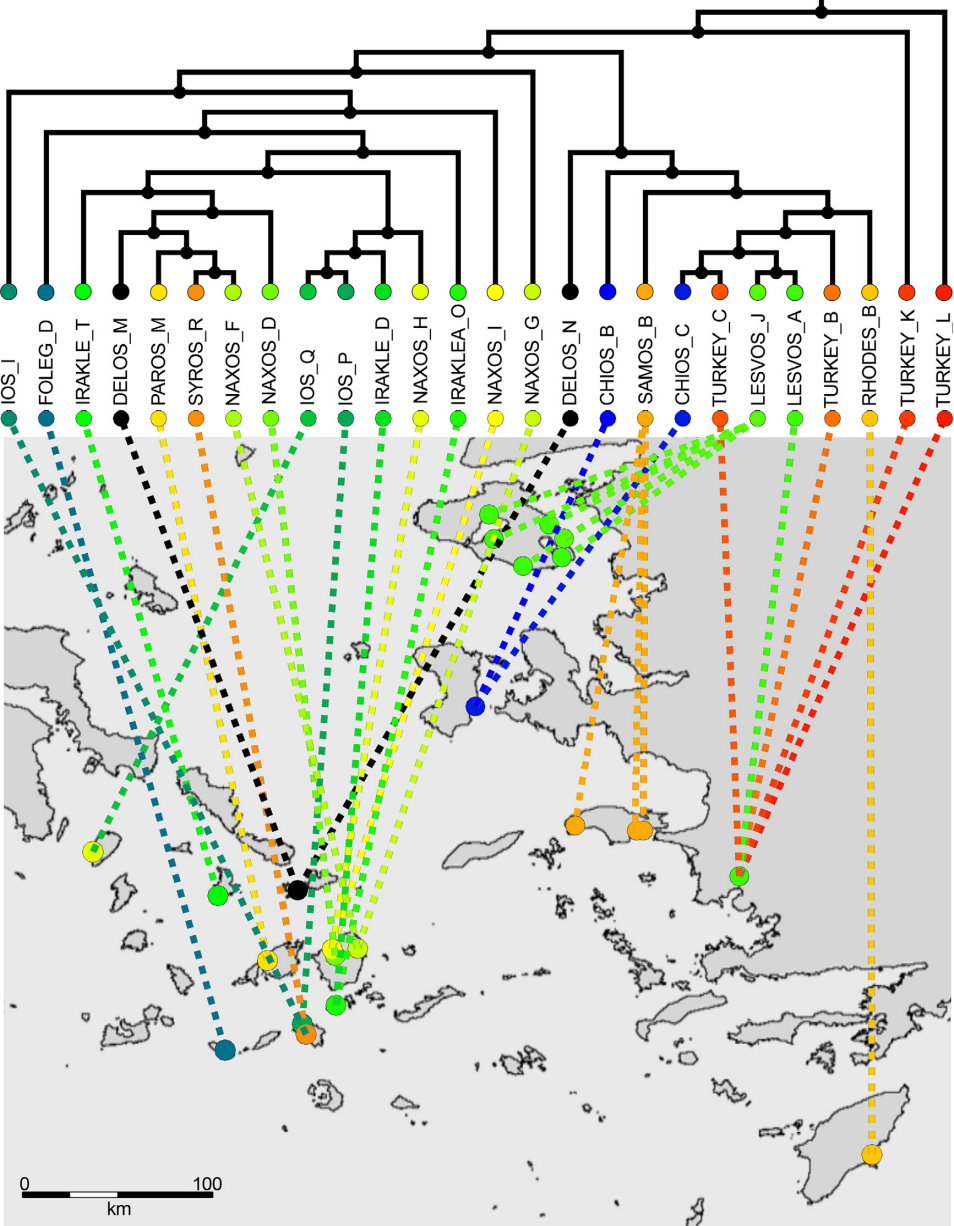
*Merodon* aff. *albifasciatus*2. Neighbor‐Joining tree with terminal tips placed at geographical localities of samples.

**Figure 18:**
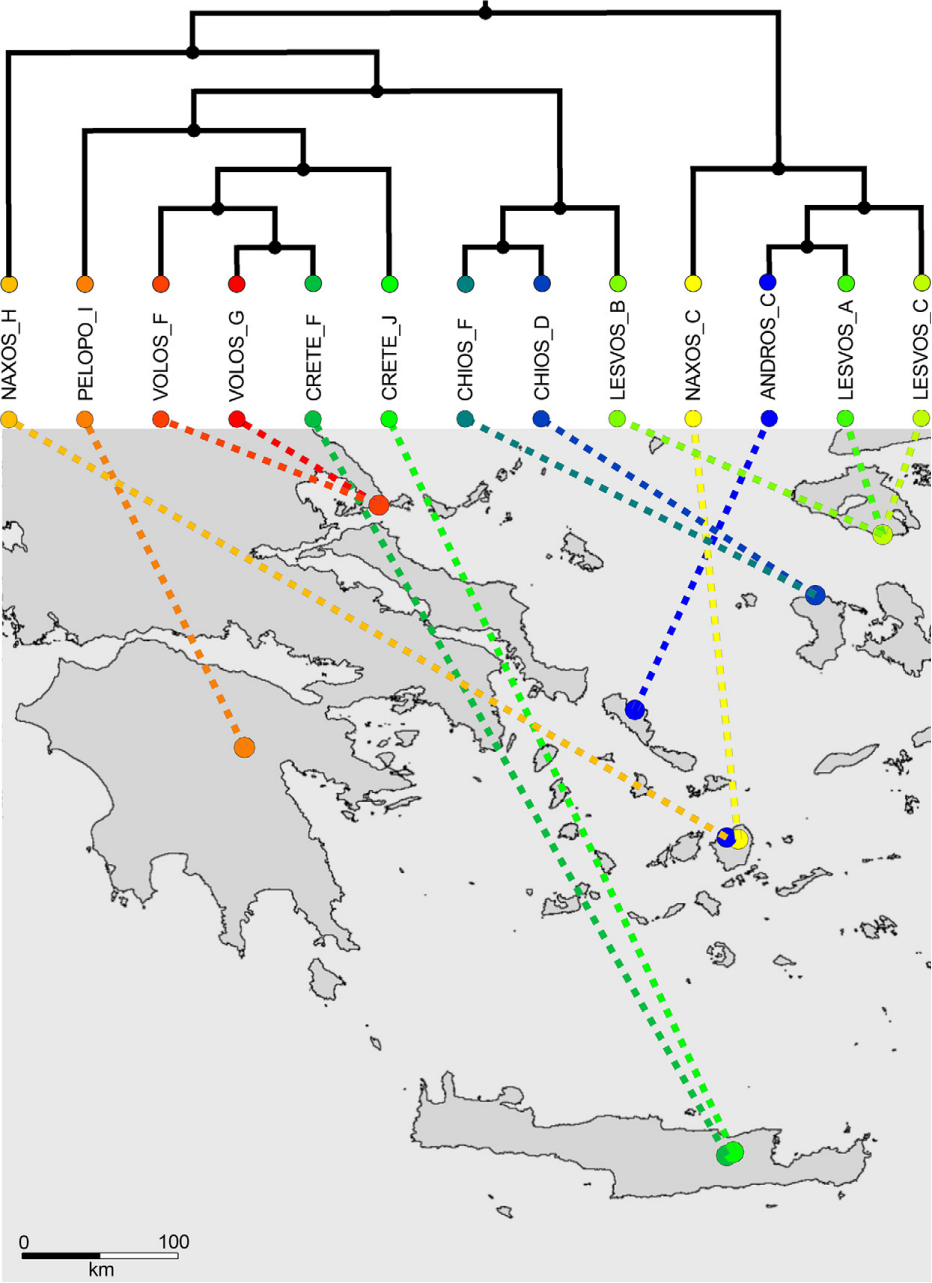
*Merodon aurifer*. Neighbor‐Joining tree with terminal tips placed at geographical localities of samples.

**Figure 19:**
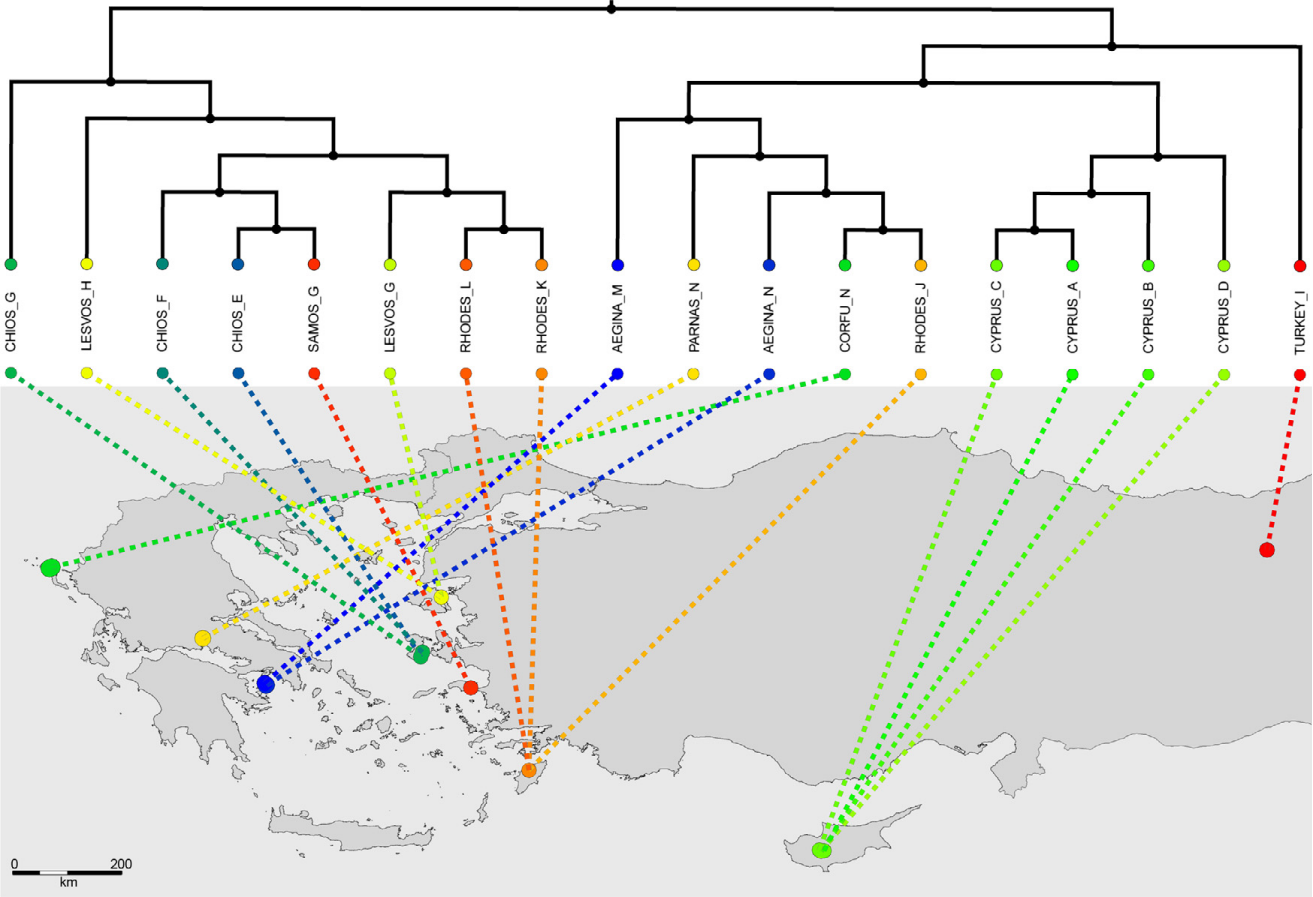
*Merodon pruni*. Neighbor‐Joining tree with terminal tips placed at geographical localities of samples.

**Figure 20:**
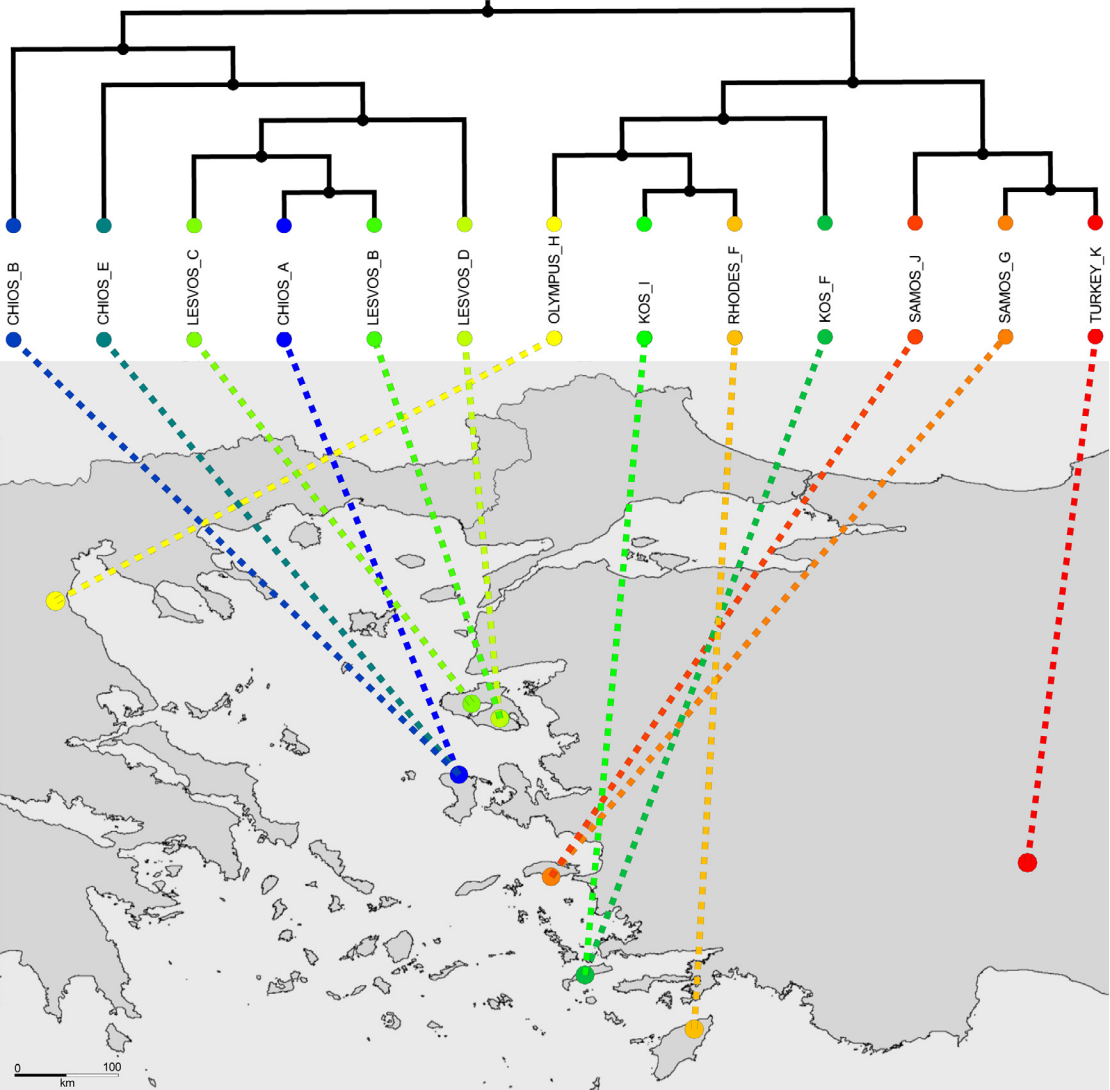
*Merodon spinitarsis*. Neighbor‐Joining tree with terminal tips placed at geographical localities of samples.

The nucleotide divergences of the mtDNA COI sequences of all species are low but clearly structured in the studied geographical space. Statistical parsimony network analyses of ancestral haplotype probabilities were congruent with the zoogeographic classification of the species established in Vujić et al. (2016), meaning that species that Vujić et al. (2016) classified as having a Balkan (*Merodon albifasciatus* and *M. clavipes*) or Anatolian (*Merodon albifasciatus*2) and *M. spinitarsis*) distributional category showed the highest probabilities of the ancestral haplotypes for samples from within the respective “source areas” of the taxa (Figs. 2, 3, 4, 5, 7, 8, 9, 10, Table 1). For the abundant and widely distributed species *M. albifrons,* the ancestral haplotype was shared on all mainland areas and a large number of islands, in agreement with its zoogeographic classification (widely distributed) (Fig. 11). Vujić et al. (2016) recorded the taxon from 19 of the 35 studied islands in Eastern Mediterranean. *M. albifrons* is the only bivoltine species among the studied taxa, and we sampled both the spring and autumn generations with some shared haplotypes among the different generations.

The widely distributed species *M. aurifer* shares the ancestral haplotype between Andros and Naxos W of MAT, and Lesvos Island E of MAT, while other collected haplotypes are either from W or E of MAT (Figs. 12, 18). Samples from Crete and Volos shared one haplotype, and had closest connection to a unique haplotype found in Peloponnese, in addition to single haplotypes present in all three localities.

The ancestral haplotype estimates differed for the four species classified in the category “Continental and large island species.” The ancestral haplotype of *M. erivanicus* was estimated as occurring on Peloponnese (Fig. 13); of *M. italicus* on Lesvos, Samos, and W Turkey (Fig. 14); of *M. pruni* on Chios, Lesvos, and Samos (Fig. 15); and of *M. velox* on Chios, Ikaria, and W Turkey (Fig. 16). The zoogeographic distributional category of the first mentioned species is Balkan and of the three last mentioned species Anatolian (Vujić et al. 2016), fully agreeing with statistical parsimony estimates of geographical locations of the ancestral haplotypes (Table 1). Assuming that the current geographical distribution of ancestral haplotypes offers a signature of “first or early existence,” will the ancestral haplotypes indicate the geographical origin of the species.

The zoogeographic classification of Vujić et al. (2016) could thus be refined to integrate the distributional category “Continental and large islands” into the Balkan and Anatolian categories as follows: (1) Balkan species, encompassing species occurring (a) on Balkan and on all sized islands and (b) on Balkan and larger islands; and, (2) Anatolian species, occurring on (a) on Anatolia and on all sized islands and (b) Anatolia and larger islands.

## Discussion

### Phylogeographic signature in *Merodon* spp.: is MAT a distributional barrier?

Poulakakis et al. (2015) stated that the diversification of terrestrial organisms in the Aegean is much more recent than the beginning of the history of this region, which started in middle Miocene with the formation of the MAT (~12–9 Mya). Results of the phylogenetic *β*‐diversity analysis (Table 2) identified the MAT as a strong past barrier whose effects are still visible today in the phylogenetic history of the clade in the Aegean area.

**Figure 21:**
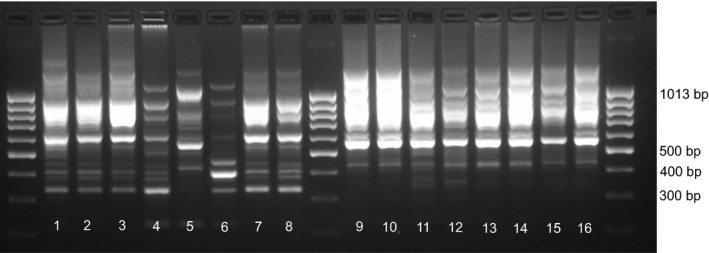
ISSR genome fingerprint of samples of *Merodon* aff. *albifasciatus*2 from localities West (lanes 1–8) and East (lanes 9–16) of the MAT. Lanes 1 & 2: Naxos, 3: Folegandros, 4: Ios, 5 & 6: Delos, 7 & 8: Irakleia, 9 & 10: Chios, 11 & 12: Samos, 13 & 14: Lesvos, 15 & 16: Turkey.

Of the five species that are distributed across the MAT (Table 1, Figs 9, 11‐14), the species *Merodon albifasciatus*2 and *M. erivanicus* show exclusively different haplotypes on islands situated E and W of the MAT (Figs. 9, 13). The genomic fingerprinting for *Merodon albifasciatus*2 was sensitive enough to intraspecifically identify specimens according to their geographical origin, a ca 330‐bp‐sized band is present in samples West of the MAT (Fig. 21 lanes 1–8, except lane 7) but not in Eastern samples (Fig. 21 lanes 9–16). *Merodon aurifer* exhibits COI haplotypes that are partly shared across the MAT (Figs. 12, 18).

### Congruence of zoogeographic and phylogeographical patterns

Most species were obtained across their entire distributional area in the Eastern Mediterranean (Table 1). Vujić et al. (2016) tested the efficiency of the species sampling effort of *Merodon* spp. in the Aegean using species accumulation curves (plotting cumulative species numbers against collecting days) for each island, and found that collections should be close to complete. The calculated parsimony networks show mean genetic divergence of 0.1–0.6% between haplotypes occurring on different islands across the Aegean region (Figs. 7, 8, 9, 10, 11, 12, 13, 14, 15, 16). Using the mtDNA mutational rate for invertebrates of 2.3% differences per million years (Brower et al. 1994), the observed mutational differences equal ages roughly of 44,000–260,000 years. The *Merodon* species are thus among the “new colonizers” (sensu Poulakakis et al. 2015) that dispersed in the region (repeatedly) in Pleistocene. Our results thus showed that mtDNA COI reflects a Pleistocene phylogeographic history of the *Merodon* species in the Aegean archipelago.

Despite the expectation of phylogeographic structure being inversely correlated with physiological (and anatomical) capacity for dispersal, the studied *Merodon* spp. presented geographically clearly structured haplotype networks. Of the fly taxa hitherto used in phylogeographic studies (viz. Simuliidae (Pramulid et al. 2005), Chironomidae (Krosch 2011), Drosophilidae (Pfeiler and Markow 2011), Neriidae (Pfeiler et al. 2013)), hoverflies of the genus *Merodon* definitively possess the largest body size and wing span, and thus, the taxon constitutes a significant contradiction to the expected negative correlation between dispersal ability and strength of phylogeographic patterns. The recent study by Vargas et al. (2015) also revealed an unexpected contradiction to general expectations, as the Carpenter Bee (*Xylocopa darwini*) showed restricted colonization success across the sea barriers of the Galapagos Islands despite possessing a very strong flight capability and a wide ecological amplitude.

Zoogeography describes the present distributions of species, with the time window covering about a hundred years of available zoogeographic distributional information based on samples of genus *Merodon* species deposited in museum collections and other repositories. The present phylogeographic study provides a different time window into the geographical distributions of *Merodon* spp., as the depicted haplotype divergences have occurred within a magnitude of a few tens of thousands or hundreds of thousands of years. All species of the present study co‐dispersed at a higher or lower extent in the Aegean area in the most recent geological period, the Pleistocene. The time estimates for the phylogeographic networks of the focal *Merodon* spp. in the Archipelago are in concordance with the hypothesis of the lower sea levels during Pleistocene glacial periods when island sizes increased, creating areas of shallow waters with small islands as connecting stepping stones.

We here establish a (new) connection between these different disciplines of historical biogeography and their different time windows, as we found that the statistical parsimony network predictions of the ancestral haplotypes for the different species coincided well with the independently assessed zoodistributional categories of these species in their Mediterranean climate regime. The distribution patterns of the species have apparently remained unchanged since dispersals took place in early or late Pleistocene at periods when sea levels were considerably lower than the present ones.

### Additional spatial patterns: Crete is more closely connected to West than East

We obtained samples from Crete for *Merodon aurifer* and *M. clavipes*. For *Merodon aurifer,* samples from Crete were closest to samples from Volos, and for *M. clavipes,* the samples from Crete are connected to samples from Naxos (Figs. 8, 12, 18). The final break‐up of the land connections between Crete and Peloponnese has been estimated at around 5 Mya (Meulenkamp et al. 1988), and some authors support a partial connection of the western part of Crete with Peloponnese during the Pleistocene (e.g., Azzaroli 1977; Lanza and Vanni 1987), a pattern that could fit the results of the present study. The formation of the MAT dividing line contributed to the separation of Crete from southeastern Aegean islands and the Anatolian Peninsula. The sea level was −120 m during late Pleistocene, *ca* 20,000 years ago, facilitating an island or stepping stone connection between Crete and the Western Aegean, but not to Eastern Aegean across the MAT.

### Unintentional human‐mediated transfer of samples?

For *Merodon albifasciatus*2, *M. erivanicus,* and *M. italicus,* we found closest haplotype connections between islands near to Greek mainland or sites on Greek mainland with haplotypes of Anatolian samples. For *M. erivanicus,* these were the only samples that were obtained for the species from Turkey (Fig. 13), but a closest haplotype connection to Greek mainland (Malesina, Mt Parnas) samples seems less likely than a closest haplotype connection of Turkish samples to samples from Anatolian seaboard islands Lesvos, Samos, and Rhodes. For *M. italicus,* a single haplotype shared between samples from Rhodes and Turkey (near Marmaris) was closest connected to a sample from Aegina Island, while other samples from Turkey (Izmir and Isparta) were closest connected to, for example, Lesvos and Samos (Fig. 14), as could be expected.

For *Merodon albifasciatus*2, a haplotype found on island of Delos was most similar to haplotypes from Turkey and Anatolian seaboard islands Chios, Samos, and Rhodes, indicating an Anatolian origin (Figs. 9, 17 haplotype Delos_N). The genomic fingerprinting supported this finding, as the genotype (Fig. 21 lane 5) showed banding pattern identity with the Eastern samples and not with Western as could be expected. The COI haplotype of the other studied sample from Delos (Fig. 17 Delos_M) was most similar to other haplotypes from localities West of the MAT. Genomic fingerprinting (Fig. 21 lane 6) showed a unique banding pattern for this sample, not clearly related with Eastern nor Western samples.

For *Merodon spinitarsis,* one single specimen was found from the Greek mainland (Olympos mnt.), while the species is not found elsewhere on Balkan mainland or western Aegean Islands. The COI haplotype showed high similarity to haplotypes registered from Kos and Rhodes (Figs. 10, 20). For *Merodon pruni,* one haplotype registered from Rhodes is most similar to other samples from Greek mainland localities (Figs. 15, 19), and not with samples from other Anatolian seaboard islands.

A plausible explanation for these deviating haplotype connections is the unintentional transfer of soil samples or plants (or parts of plants) by humans, with larvae or pupae of the discussed species encapsulated within the transferred material, eventually resulting in the establishment of the “aberrant” haplotype in the new area. As the host plants of the *Merodon* flies belong to a plant group that has been utilized at least since the antiquity, such an unintentional human‐mediated transfer of the flies could, indeed, occur. Plant bulbs were eagerly sought after for people's everyday use and well‐being: there is evidence (cf. Gennadios 1914; Kavvadas 1956), that in the antiquity many bulbs belonging to *Muscari (comosum?), Crocus sativus, Urginea maritima, Pancratium maritimum, Ornithogalum* spp.*, Narcissus, Orchis, Serapias, Arum, Arisarum, Asphodelus*, and *Iris* have been widely used as foodstuffs, condiments, and dyes [several taxa inferred by Theophrastus (*Historia Plantarum*, book 7), Athenaeus (*Deipnosophistae* books 1–15), and Dioscorides Pedanius (*de Materia Medica,* book II], as medicaments (Dioscorides Pedanius, *de Materia Medica,* book II; Arnott 1999), or as perfumes (Martlew 1999).

Given the long history and wide use of bulbous plants especially in the kitchen and in medicine, it will not be surprising if some plants were traded through the Aegean, probably originating from the Near or the Middle East. Such trading routes can be revealed through readings of the Greek Scholars, as the description by Dioscorides Pedanius (40–90 AD) of 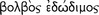 (the edible bulb, i.e., *Muscari comosum* according to Kavvadas 1956 and Gennadios 1914), of which “the red” type was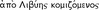, “ ”, i.e. imported from Libya. An important trade place for many centuries in the ancient Greek World was Delos, which together with its sacred character resulted in the island being for a long time extremely important connecting point between the Levant and the West (Encyclopaedia Drandakis). Aegina, on the other hand, has been firstly occupied/inhabited by immigrants originated from Asia Minor, probably from Karia (an area opposite to Samos and Rhodes), and repeated times inhabited by Phoenicians (Encyclopaedia Drandakis).

Similar cases have been inferred by Poulakakis et al. (2015) who reported that the human‐aided herpeto‐faunal dispersal has altered the distribution patterns of several species in the Aegean area. The authors further discussed the possibility of human/accidental translocation of terrestrial invertebrates such as *Cicada* spp. (cicadas are usually associated with olive trees) and the dispersion of the spider *Eresus walckenaeri* in wooden material.

## Conclusions

Our results identified that interisland distance is partly responsible for the observed haplotype distributional patterns, namely the replacement of haplotypes, and the MAT as a strong past barrier, the effects of which are still visible today in the phylogenetic history of the clade in the Aegean. Based on this, we infer that the sea still acts as efficient dispersal barrier for *Merodon* species. During the Pleistocene glacial periods, sea levels have varied considerably, from 200 m (Riss, 250–125,000 ya), 120 m (Würm, 75–21,500 ya), to 60 m (11,500 ya) lower than present (Perissoratis and Conispolatis 2003; Comes et al. 2008). Thus, during the last glaciations, the low sea level (−120 m vs. present) allowed the connection between the Northern Sporades Islands with the Greek mainland; some eastern Aegean Islands (including Lesvos, Chios, and Samos) with Asia Minor; and the interconnection of most of the Cycladic Islands (Perissoratis & Conispolatis 2003). At the beginning of the Holocene (*ca* 11,500 ya), the sea level rose 60 m, and, consequently, most of the Aegean Islands were disconnected from each other and the adjacent mainland(s). Based on the Pleistocene sea level changes, the Aegean Islands were likely subjected to multiple separate colonization events of *Merodon* spp. Many Balkan elements could have easily reached Cyclades and Northern Sporades, but not the Southern Sporades, because of the persistence of the sea barrier. In contrast, Anatolian species could have easily reached the eastern islands, but not those of the central Aegean and the western ones, as also outlined by Fattorini (2002). In addition to the events resulting from climatic oscillations during Pleistocene that shaped the organismal history of the region and the uniqueness of the area from a zoogeographic point of view as a melting point of several different faunas, we can also add that the area is unique also from a fly phylogeographic perspective as shown in the present study. Thus, there is not only a melting point region, but also a meeting point region.

## Data accessibility

GenBank accession numbers for the COI barcode are presented in online Supplementary Information S1. [Previously generated COI barcodes from Lesvos (Ståhls et al. 2009 are *M. albifrons* (FM206495 and FM206510), *M. erivanicus* (FM206487 and FM206512), *M. pruni* (FM206498), *M. spinitarsis* (FM206489 and FM206505), and *M. velox* (FM206506‐09 and FM206496)]. MtDNA COI sequence data matrix uploaded as Supporting Information S2.

## Conflict of Interest

None declared.

## Supporting information


**Table S1.** Data on specimens used for molecular work.
